# Expression patterns of *SLIT*/*ROBO* mRNAs reveal a characteristic feature in the entorhinal-hippocampal area of macaque monkeys

**DOI:** 10.1186/s13104-020-05100-7

**Published:** 2020-05-27

**Authors:** Tetsuya Sasaki, Yusuke Komatsu, Tetsuo Yamamori

**Affiliations:** 1 Division of Brain Biology, National Institute for Basic Biology, 38 Nishigonaka Myodaiji, Okazaki, 444-8585 Japan; 2 Present Address: Department of Anatomy and Neuroscience, Faculty of Medicine, University of Tsukuba, 1-1-1 Tennodai, Tsukuba, Ibaraki 305-8577 Japan; 3 Ph.D Program of Neurosciences, Graduate School of Comprehensive Human Sciences, University of Tsukuba, 1-1-1 Tennodai, Tsukuba, Ibaraki 305-8577 Japan; 4 Laboratory of Veterinary Hygiene, Graduate School of Veterinary Medicine, Hokkaido University, Sapporo, 060-0816 Japan; 5 Laboratory for Molecular Analysis of Higher Brain Function, RIKEN Center for Brain Science, Wako, Saitama 351-0198 Japan

**Keywords:** Axon guidance, Cerebral cortex, Entorhinal cortex, Hippocampus, In situ hybridization, Primates

## Abstract

**Objective:**

SLITs are secreted glycoproteins that bind to Roundabouts (ROBOs) which are a family member of transmembrane receptors. SLIT signaling has well-conserved roles in mediating axon repulsion in a developing nervous system. We previously reported that *SLIT1* mRNA is enriched in middle layers of the prefrontal cortex of macaque monkeys in a developmentally regulated manner. Other *SLIT* (*SLIT2* and *SLIT3*) mRNAs showed preferential expressions in the prefrontal cortex with a distinct laminar pattern. To obtain further clues to the role of SLIT signaling in the organization of the primate brain, we performed ISH analysis of *SLIT* and *ROBO* mRNAs using adult macaque brain tissues.

**Results:**

In this study, we examined the expression patterns of *SLITs* and *ROBOs* (*ROBO1* and *ROBO2*) in other brain regions, and found intense and characteristic expression patterns of these genes in the entorhinal-hippocampal area. In situ hybridization analysis revealed that *SLIT1* and *SLIT2* mRNAs showed marked complementary distribution in the entorhinal cortex. *SLIT* and *ROBO* mRNAs were widely expressed in the hippocampus with modest regional preference. These findings suggest that each *SLIT* gene has a specialized role that is particularly important for prefrontal as well as hippocampal connectivity in the primate cortex.

## Introduction

SLIT is a repellent guidance molecule, which is well conserved in various species [1, 2]. The repellent effect of SLIT is mediated by the receptor, Roundabout (ROBO) [3]. To date, three *Slit* genes, *Slit1*-*3* [4] and four *Robo* genes (*Robo1*-*4*) have been identified in vertebrate genome [3, 5–7]. In mammals, SLIT/ROBO signaling is reported to have essential roles in the development of the nervous system including midline crossing, as observed in *Drosophila* [8, 9] and formation of major axonal tracts [10–13]. Furthermore, recent studies demonstrate expanded functional repertories of SLITs and ROBOs, such as neurogenesis, cell proliferation/migration, angiogenesis, oncogenesis, and involvement in several diseases [2, 14–17].

We have investigated the molecular basis of differences in the architecture across neocortical areas and identified three genes, *SLIT1* [18], *RBP4* [19], and *PNMA5* [20] that are highly expressed in the higher-order association areas of macaque monkeys [21]. Among them, *SLIT1* mRNA in particular is preferentially expressed in the prefrontal cortex compared with other association areas. Our detailed *in situ* hybridization (ISH) analysis demonstrates that *SLIT1* mRNA is mainly distributed in the middle layers of most cortical areas, highest in the prefrontal cortex but lowest in the primary sensory areas. The prefrontal-enriched pattern was established by reduced expressions, specific for areas and layers during postnatal development. The promoter region of *SLIT1* gene is hypermethylated, and it is assumed that some regulatory elements (e.g., methyl-binding proteins) are involved in the area selective expression [22]. Other *SLIT* (*SLIT2* and *SLIT3*) mRNAs showed modest preference in the prefrontal cortex, whereas *ROBO1* and *ROBO2* mRNAs were widely detected within the cerebral cortex. Since cortical neurons, particularly those in the prefrontal cortex, simultaneously express *SLIT1* and *ROBO* mRNAs, SLIT1 could work in either an autocrine or paracrine manner in the postnatal primate cortex, which implies that it has other functions in addition to its role as guidance cues.

In this report, to obtain further clues to the role of SLIT signaling in the organization of the primate brain, we performed ISH analysis of *SLIT* and *ROBO* mRNAs using adult macaque brain tissues. We examined the expression patterns of these genes in detail in brain regions other than the prefrontal cortex. We found unique expression patterns of these genes in the entorhinal-hippocampal area.

## Main text

### Materials and methods

#### Experimental animals

For ISH experiments, brains from five macaques (*Macaca fuscata*, juvenile to young adults, 2.6, 4.0, 5.2, 5.6, and 5.8 years old) were used. The animals were anesthetized and the brain fixed as described previously [18]. Sections from the blocks that included the entorhinal cortex and hippocampus were sliced at 35 µm thickness (Additional file 1: Figure S1).

#### In situ hybridization

The cDNA fragments were obtained by RT–PCR using the primers listed in Table 1, and subcloned into the pBlueScriptII vector. The concentrations of all the riboprobes used in this study were adjusted to 0.1 μg/μl and the riboprobes were stored at − 30 °C. Single-color ISH was carried out essentially as previously described [18, 23]. We used more than two types of probe for each of *SLIT1*, *SLIT2*, *ROBO1*, and *ROBO2*, and confirmed that each probe exhibits the same pattern of signal distribution (data not shown). After the initial confirmation, multiple probes were mixed together to enhance ISH signals. We also confirmed that the sense probes detected no more signals above the background level. The layer positions of entorhinal cortex and the boundaries with hippocampal fields were determined on the basis of Cresyl violet staining of adjacent sections. Cresyl violet staining shows clear differences in cell size and packing density among the layers in the entorhinal cortex and the four CA fields in hippocampus [16, 24, 25].

**Table 1:** ISH probes used in this study

Gene name	Probe name	Species	Accession No.	PCR primer set	Length
*SLIT1*	Slit1-1	macaque	NM_003061	cttccaggacctgcagaacc	552
				cccgtcttcgatctcggaca	
	Slit1-2	macaque	NM_003061	aagtttgaatgccaaggtcc	448
				actgggcctcgtgttgacat	
	Slit1-3	macaque	NM_003061	cttgtgctctccggatctga	822
				gtacaggtttcggatgcaac	
	Slit1-4	macaque	NM_003061	cctgtggcagatcctcaacg	647
				ccatcgctgcactcaaaggt	
*SLIT2*	Slit2-1	macaque	NM_00478	cccaggaatatcccccgcaa	770
				gagaccatcacagaaatacg	
	Slit2-4	macaque	NM_004787	cagcccctgtgataattttg	866
				gtcctctgtgatgaagagga	
*SLIT3*	Slit3-3	macaque	NM_003062	ttgacctgagcaacaacagc	838
				ccctggacaaaggattcag	
*ROBO1*	Robo1-1	macaque	NM-022188	ggagaggctgtgagccacaa	942
				tcctgtgaatcagactgtag	
	Robo1-3	macaque	NM-022188	tggttagtttttgaagtgag	877
				acctacagtcgcccagctga	
	Robo1-4	macaque	NM-022188	ctgatgctccctgagtcaac	868
				ggctacatttcaggacccct	
*ROBO2*	Robo2-1	macaque	NM_002942	aggaactatcttggtgaagc	700
				ggaaacccacagccagctgt	
	Robo2-4	macaque	NM_002942	ccaggccaaggggataaaac	673
				gcctatcagtttgatatagc	

We confirmed that the multiple probes for one gene exhibit the same distribution pattern. After the initial confirmation, these multiple probes were mixed to enhance the ISH signals

#### Image analysis

**Fig. 1:**
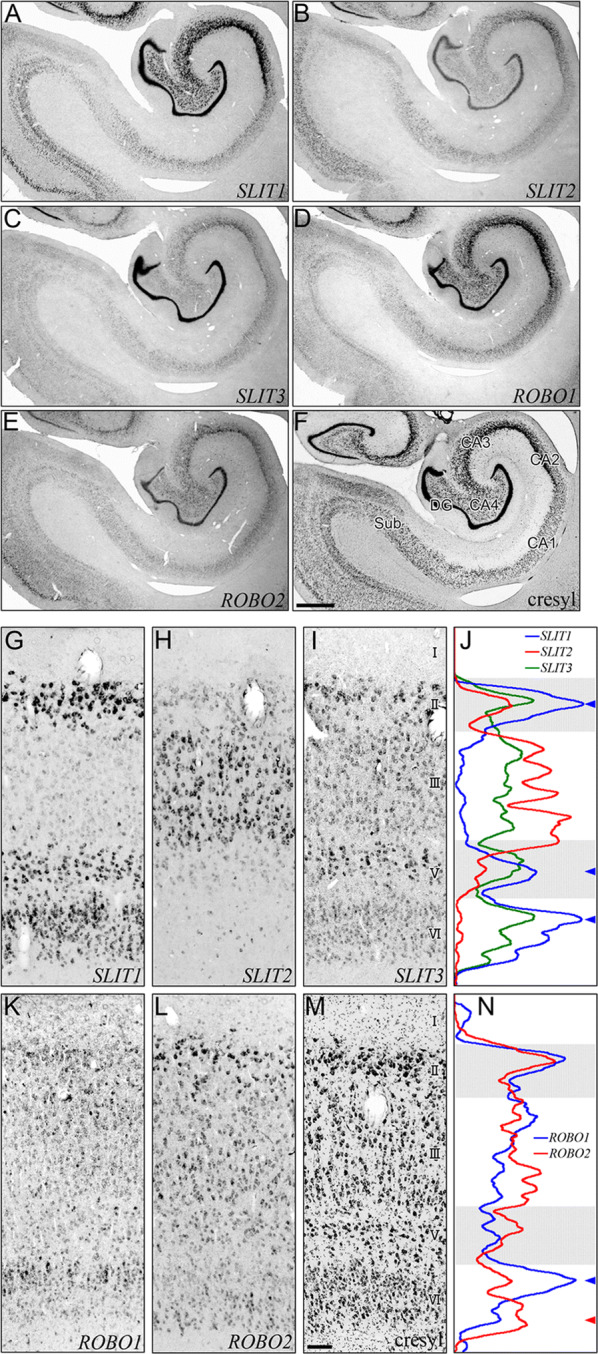
Expression of *SLIT* and *ROBO* mRNAs in Hippocampus and Entorhinal Cortex. In situ hybridized sections for detection of *SLIT1* (**a**), *SLIT2* (**b**), *SLIT3* (**c**), *ROBO1* (**d**), and *ROBO2* (**e**), and cresyl violet-stained section (**f**) of the hippocampus. DG: dentate gyrus; Sub: subiculum; ProS: prosubiculum; PrS: presubiculum. Scale bar = 1 mm. Layer distributions of *SLIT1* (**g**), *SLIT2* (**h**), *SLIT3* (**i**), *ROBO1* (**k**), and *ROBO2* (**l**) mRNAs in entorhinal cortex. Adjacent section for cresyl violet staining (**m**). Scale bar = 100 µm. The laminar profiles indicate the layer distributions of *SLITs* (**j**) and *ROBOs* (**n**). The density profiles of *SLIT1* (blue), *SLIT2* (red) and *SLIT3* (green) were plotted along cortical depth (**j**). The density profiles of *ROBO1* (blue) and *ROBO2* (red) were plotted along cortical depth (**n**)

Eight-bit gray scale color images were obtained using the digital color camera DP 70 (Olympus, Tokyo, Japan) attached to a BX-51 microscope (Olympus). The background image was subtracted using Image-Pro Plus image analysis software (Media Cybernetics, Silver Springs, MD). The laminar distribution patterns of different ISH signals (Fig. 1j, n) were analyzed as previously described [18]. Signals were extracted from the background component by converting the eight-bit gray-scale images into the binary images. The threshold used here was set to the standard deviation (SD) beyond the average intensity of each cortical section. Then, we calculated average values with respect to each row to obtain the line profile in regions of interest (ROIs: 100 µm bin, which is the height of the cortex from layer I to WM) using Image Pro Plus.

### Results and discussion

SLITs/ROBOs are reported to be involved in the formation of hippocampal connections during the rodent development [26]. We examined the *SLIT* and *ROBO* mRNA expression patterns in the hippocampus and its surrounding areas in the adult macaques (Additional file 1: Figure S1).

In and around the hippocampus, all the *SLIT* and *ROBO* mRNAs were widely expressed (Fig. 1a–f). Each gene showed moderate regional preference. For example, the most intense signals of *SLIT1* and *ROBO1* mRNAs were observed in the granule cell layer of the dentate gyrus (DG, Fig. 1a, d), followed by the expression in the pyramidal cell layer of cornus ammmon (CA) 2-CA4. The expression levels of *SLIT1* and *ROBO1* mRNAs were rather low in CA1 and the subiculum. *SLIT2* and *ROBO2* mRNAs were also highly expressed in the DG and uniformly distributed in CA1-CA4 (Fig. 1b, e). *SLIT3* mRNA was mainly expressed in the DG and relatively weak in other regions (Fig. 1c).

The entorhinal cortex is the interface between the hippocampus and the neocortex [27, 28]. It contains grid cells with their unique firing discharge pattern, and was reported as the key area for spatial representation in mammals [29, 30]. Abnormalities in the cells of layer II of the human entorhinal cortex have been implicated in the pathophysiology of schizophrenia and Alzheimer’s disease [31, 32]. In the entorhinal cortex, Densitometric analysis indicated conspicuous laminar preference of *SLIT* and *ROBO* mRNAs. Most strikingly, *SLIT1* and *SLIT2* mRNAs showed complementary distribution (Fig. 1j). Intense signal of *SLIT1* mRNA was observed in layers II, V, and VI (Fig. 1g and j, blue arrowheads) with only a low signal intensity of *SLIT1* mRNA in layer III. Large cells that constitute the characteristic cell islands in layer II [16] showed a particularly high signal intensity of *SLIT1* mRNA. On the other hand, *SLIT2* mRNA was predominantly expressed in layer III (Fig. 1j), with only a weak expression in other layers (Fig. 1h). This complementarity is reminiscent of the pattern in area TE except that *SLIT1* and *SLIT2* mRNAs are expressed in different layers [18]. Owing to the different laminar preferences of these genes, we observed a sharp border between the entorhinal cortex and the perirhinal cortex (data not shown). *SLIT3* mRNA was expressed similarly across layers in the entorhinal cortex, although intense signals were observed in the layer II and the upper part of layer V. *ROBO1* and *ROBO2* were also expressed widely across layers, which is similarly observed in other cortical areas. Interestingly, *ROBO1* and *ROBO2* mRNAs were abundant in the upper and lower parts of layer VI, as indicated by blue and red arrowheads in Fig. 1n, respectively, but the pattern not in neocortical areas. The differences in the laminar distribution among the *SLIT*s in both the entorhinal cortex and neocortex might reflect different functions of these genes in the cerebral cortex of primates.

Although the roles of SLITs/ROBOs in the guidance of neurons during development may be expected, their functions in postnatal brains, where no major guidance events occur, remain unclear. A possible role of these molecules is in the control of regeneration in response to injury. All *Slits* are reported to be expressed in reactive astrocytes at the injury site portion of the rat brain [33]. Since *ROBO* mRNAs are expressed in substantial cell populations in the cerebral cortex, SLITs secreted around the injury site may inhibit irrelevant axonal regeneration. Another plausible possibility is that the SLIT-ROBO system has a role in maintaining certain neuronal morphologies and circuits [34–37]. Lines of evidence suggest that axon guidance molecules are implicated as critical regulators in synaptogenesis and synaptic plasticity [37–39]. Numerous studies have shown the dynamics of neuronal processes in the postnatal cortex [40–42]. In this regard, we note that *SLIT* mRNAs were abundant in the brain regions where high neuronal integration and/or plasticity plays roles, such as the entorhinal cortex, dentate gyrus, and prefrontal cortex, where the other higher-order association area enriched genes, *RBP4* and *PNMA5* are also highly expressed [19, 20]. These genes may subserve neural plasticity and cognitive function. Further research is needed to examine these possibilities.

## Limitations

In this study, we found the characteristic expression pattern of the axon guidance molecule SLITs and its receptor ROBOs in the hippocampus and entorhinal cortex of adult macaque monkeys. We did not perform other methods such as qPCR or immunohistochemistry. Gene manipulation analysis including overexpression and suppression of gene expression will be needed to test our hypothesis that these groups of molecules are involved in structural plasticity of postnatal primate brains.

## Supplementary information

**Additional file 1: Figure S1.** A coronal section of macaque brain containing hippocampus and entorhinal cortex for cresyl violet staining. Scale bar = 5 mm. *EC* entorhinal cortex, *HC* hippocampus, *LGN* lateral geniculate nucleus, *TEd* dorsal inferotemporal cortex, *TEv* ventral inferotemporal cortex, *sts* superior temporal cortex, *PC* perirhinal cortex, *D* dorsal, *V* ventral, *L* lateral, *M* medial.

## Data Availability

The datasets, which were used and/or analyzed in the current study, are available from the corresponding author on reasonable request.

## Abbreviations

CA: Cornus ammmon
DG: Dentate gyrus
ISH: In situ hybridization
ProS: Prosubiculum
PrS: Presubiculum
ROBO: Roundabouts
Sub: Subiculum
