# Supplementary figures and images for: Expression patterns of SLIT/ROBO mRNAs reveal a characteristic feature in the entorhinal-hippocampal area of macaque monkeys
Source: BMC Res Notes. 2020 May 27;13:262. doi: 10.1186/s13104-020-05100-7 (PMC7251749; doi:10.1186/s13104-020-05100-7)

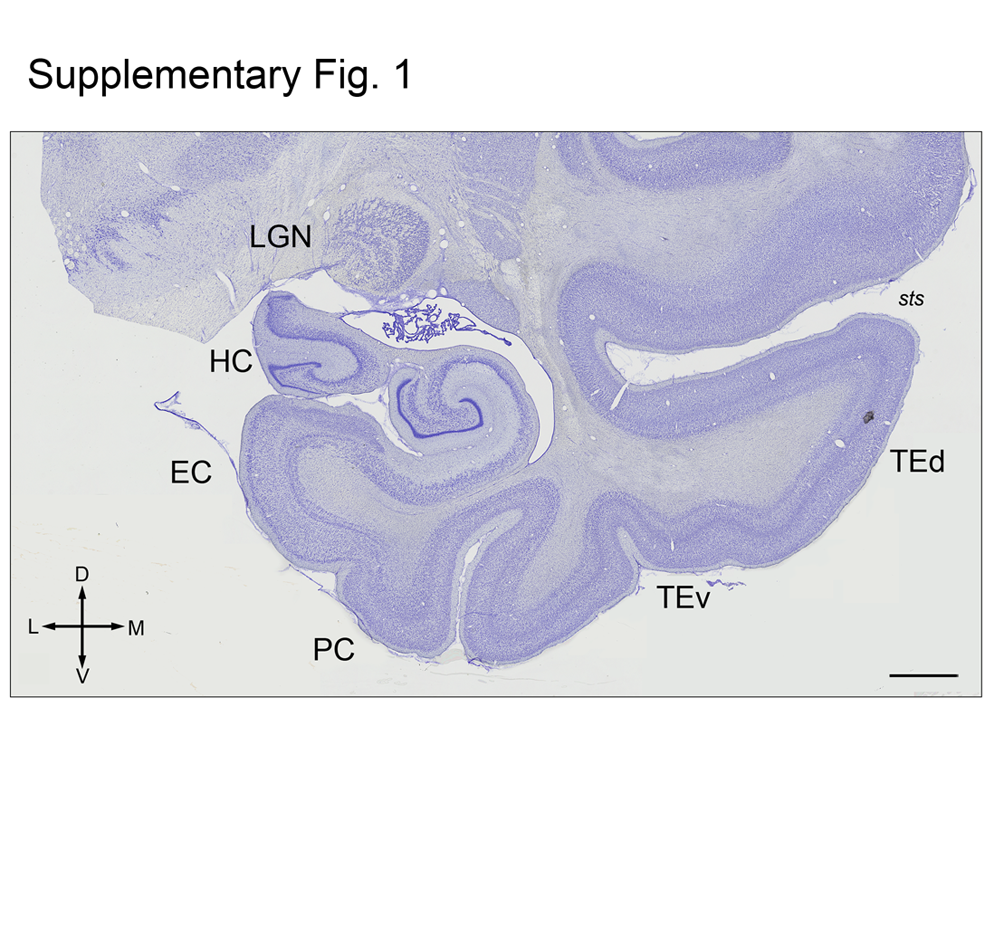

Supplement: Supplementary file 1 — Additional file 1: Figure S1. A coronal section of macaque brain containing hippocampus and entorhinal cortex for cresyl violet staining. Scale bar = 5 mm. EC entorhinal cortex, HC hippocampus, LGN lateral geniculate nucleus, TEd dorsal inferotemporal cortex, TEv ventral inferotemporal cortex, sts superior temporal cortex, PC perirhinal cortex, D dorsal, V ventral, L lateral, M medial. [file 13104_2020_5100_MOESM1_ESM.tif]
